# Supplementary material for: The neuronal chaperone proSAAS is highly expressed in the retina
Source: PLoS One. 2025 May 16;20(5):e0321867. doi: 10.1371/journal.pone.0321867 (PMC12083831; doi:10.1371/journal.pone.0321867)
Supplement: S1 File — (PDF) [file pone.0321867.s001.pdf]

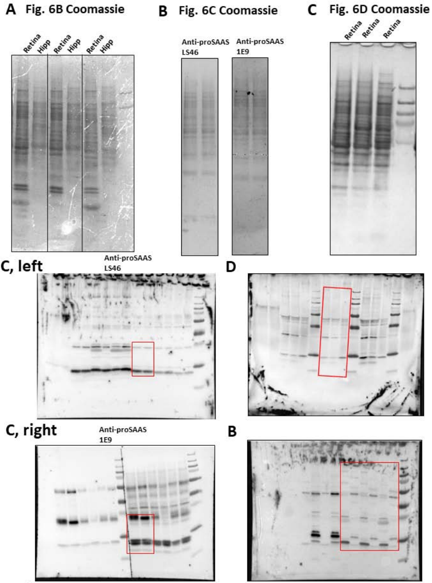

Supplementary Figure 1

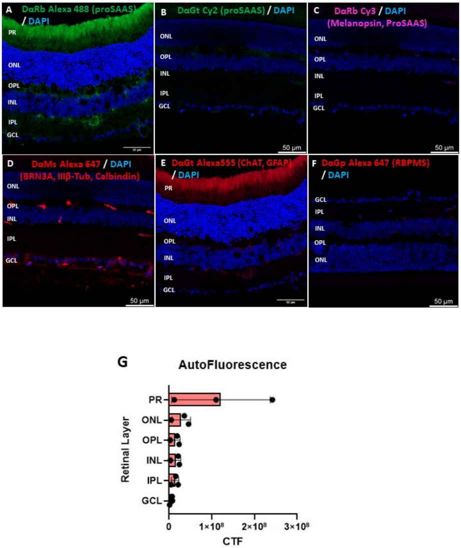

Supplementary Figure 2
