# Supplementary material for: The neuronal chaperone proSAAS is highly expressed in the retina
Source: PLoS One. 2025 May 16;20(5):e0321867. doi: 10.1371/journal.pone.0321867 (PMC12083831; doi:10.1371/journal.pone.0321867)
Supplement: Attachment — Submitted filename: PLoS rebuttal letterfinal_IL022425.docx [file pone.0321867.s004.docx]

February 26, 2025

Dear Dr. Ginsberg,

Thank you for the review of our manuscript [PONE-D-24-48169] ‘*The neuronal chaperone proSAAS is highly expressed in retinal neurons.*” We very much appreciate the positive comments of **Reviewer 1**.

With regard to the comments of **Reviewer 2**, we note that their request for “sequencing of bands from Western blots” is biochemically impossible. We also feel that their request for very expensive new animal experiments (siRNA, MERFISH) that are far outside of our expertise is both unreasonable and irrelevant to the major point of the study: that the proSAAS chaperone is abundant within the retina, as it is in hippocampal synapses (Matias et al 2024). Three lines of evidence: transcriptomics, immunofluorescent staining and Western blot experiments support this conclusion. We have, however, addressed the majority of Reviewer 2’s **33** minor concerns, and have also attempted to address his major concern of antibody specificity by adding additional language to the Methods; by referencing our recent and older papers; and by showing epitopes for all proSAAS antibodies in Figure 5 (now Figure 6). In addition, we have reformatted Figure 1 (now Figures 1 and 2) for clarity.

We appreciate the time and effort taken to evaluate our manuscript. Below is a list of the changes (shown in red both here and in the manuscript) we have made in response to the comments of the reviewers. Small wording changes to improve the manuscript are not shown.

**Reviewer #1:** This article by Schaffer et al. describes the expression pattern of the proSAAS chaperone in the mouse retina, focusing on RGCs. The manuscript is well written and the experiments are well performed. **The conclusions are consistent with the results.**
There are only a few comments.
-Brn3a+ RGCs are vision-forming RGCs and do not express melanopsin. Conversely, non-vision-forming RGCs express melanopsin (only M1-M3 subtypes express this protein at immunodetectable levels) but do not express Brn3a (for a detailed review of pan-RGC markers see PMID: 36594396). They should clarify both points in their manuscript. We have now clarified these two points in the manuscript by designating subtypes in our text.
-As pNFH is highly expressed in RGC axons, immunodetection of this neurofilament with proSAAS, which appears to be axonal, may be more sensitive than tubulin. We agree but have no more slices left to analyze, nor do we have pNFH antiserum.
-The species of the sample should be clearly indicated in the figure legends. We have now indicated the species in all figure legends.
-Finally, although not compulsory, their RGD data would improve if they analyse proSAAS expression on RGCs in mouse flatmounts. We wish we had additional animals to perform this additional experiment.
-Note: The 5xFAD mouse strain is included in the methods but not in the results. We apologize for the inclusion of the 5xFAD mice in the Materials and Methods. These data are not in the paper. We have now removed this language.

**Reviewer #2:** 

 Several critical methodological issues needs to be addressed by the authors in order to adequately verify their results. The most important is the **specificity of their utilized proSAAS antibodies.** Other concerns is stated below. We have addressed antibody specificity issues below on page 4 of this rebuttal.

- In the methods and materials section associated with the “Animals” paragraph, the author states that both wild-type and 5xFAD mice for the ICC and western blot procedures. Please state exactly how many of each phenotype that have been used as well as other associated age and gender. We include no AD mouse data in this paper. We apologize for erroneously including these mice in the Materials and Methods; this has now been removed.

Furthermore, is there a reason for pooling the retina and hippocampi of different phenotypic mice? The reviewer misunderstands. We did NOT pool retina and hippocampi from different phenotypes. We do not make any conclusions regarding our mice and AD in this manuscript. Again, there are no AD mice in this manuscript.

- In the Methods section associated with the “Human tissue”. Please state the number and phenotypic characteristics of the donor patients. Is these healthy, young patients or diseased patients with local and systemic conditions affection the retina and/or optic nerve? We have now included a description of the single healthy human donor in the Materials and Methods. As might be expected, human samples are not easy to obtain.

-Furthermore, what is the post-mortem time(s) before the retinas have been stored? How exactly have the retinas been cryopreserved? Furthermore, is there a reason why human retinas only have been used Western blotting, while IHC-analyses (which were performed on the murine retinas) have been left out? Post-mortem information is not available for this sample; we now so state. The remainder of the requested information is now available in the manuscript. We did not perform both Western and IHC on human samples because only one (pooled) human retinal sample was available, and the two techniques are incompatible.

- In the method section associated with the “meta-analysis” paragraph. On which criteria did you select the 2738 human retina samples? What log-transformation have been used? What is your scientific reasoning for removing outliers and zero-values from your analyses? Does inclusion of these values in your analysis affect the conclusion?

We thank Reviewer #2 for their input on our ARCHS4 Meta-analysis. The samples were selected based only the availability of samples in the ARCHS4 dataset of retinal tissues. The data were Log(2) + 1 transformed. To address the critique of outlier removal, we have now normalized the transcript counts using the ComBat batch correction algorithm [21] which is both more sophisticated and robust than our original method. This has allowed us to include all relevant data points in the distribution; no data are omitted.  Our original conclusions have not been altered by this change in methodology.

- Considering Figure 1A: I guess the y-axis should be proportion of samples? Your transcriptomic quantity distributions are non-normally distributed. it would more appropriate to report median and interquartile ranges.

The y-axis indeed represents the proportion of samples, and the distributions are indeed not normally distributed. We have now included references to papers discussing the relevance of gene expression distribution. The vertical lines in the density plots also now show the mean values. We have also included data from mouse samples in addition to human samples, which better aids in the comparison of studies between these two species. We recognize Reviewer’s 2 request to report the median and interquartile range (IQR); however, we feel this additional calculation would not enhance the findings from this figure since it can be reasonably extrapolated based on the distribution spread in the density plot graphs and would add unnecessary complexity to the figure.

- Consider Figure 1b: I guess the “gray quadrant” means “not detected”? please state the meaning? Furthermore, there is a huge interstudy difference in the normalized expression of proSAAS, which especially is low for Orozco and Wang. Could you identify any reasons for this? Could it be relevant to include in your discussion? Results of figure 1C could likewise be included in this discussion. Is there any reason why any statistical comparisons across the different cell-types within each study have not been performed in any of figure 1B and 1C?   We have now completely redone this figure for clarity (see new **Figure 2**). The cell-type analysis is now shown in this figure using Broad Institute software. Making statistical comparisons across cell types is not customary, as these data simply indicate the distribution of proSAAS message associated with each cell type marker.

Regarding interstudy differences, these are usually due to batch effects in sequencing. Variances due to these effects do not interfere with our conclusions which are drawn from comparing transcript expression across cell-type clusters within each study, as compared to across studies.

- Considering Figure 1d (now Figure 2d): While transcriptomic studies may indicate disturbances at the protein and functional levels, it is merely a surrogate. Hence, the proteomics studies are the only relevant studies to include in this figure if you want to argue that proteostatic dysregulation of proSAAS is present in several retinopathies. In that regard, only the glaucoma study by Mirzaei et al detects a statistically significant fold-increase of proSAAS. What kind of phenotypic glaucoma patients is this? At what disease stage(s)? Could be relevant in your discussion. The reviewer misunderstands. We do not argue that proteostatic dysregulation of proSAAS occurs. In this figure we show that proSAAS expression is associated with disease states, not that proSAAS itself is a target of the proteostatic dysregulation in these diseases. In other words, we argue (based on previous findings; see **Shakya et al 2020**), that proSAAS may potentially be upregulated in the retina to alleviate proteostatic dysregulation caused by other proteins.

We have now added a sentence to clarify this idea (and to add a caveat): “While the glaucoma proteomics data support upregulation of proSAAS levels, additional proteomics studies of proSAAS levels associated with retinal disorders involving proteostatic dysregulation are needed to confirm this positive association.”

- A general concern of mine is the specificity of the antibodies used (LS46-serum derived and the commercial 1E9). It is good practice that non-primary negative controls have been used to assess the unspecific labelling patterns of the secondary antibodies used. We totally disagree; it is not normal practice to omit primary antibodies in Western blots. This is not customarily performed because the molecular masses provide specificity (unlike in IHC).

However, this doesn’t take into account the specificity of your primary antibodies, which I fear may be low based on the results provided in Figure 2, 3, 4, 5. Could you provide evidence of the specificity of your antibodies against the different proSAAS isoforms? We discuss the antibody specificity issue on page 4 below; we are quite confident of our antibody specificity based on epitope mapping (See Figure 6!). Use siRNA for retinal knockout as a negative control? We feel that this is not a reasonable request, since it requires very expensive new animal experiments (and is indeed not customarily required for validation of a well-characterized antibody).

Determine the amino acid sequence of the protein(s) in the 13, 17, and 21 kDa bands derived from the retinal protein extracts to verify protein nature? We feel this request is unreasonable, and quite frankly, technically impossible. We could easily determine that these immunoreactive bands are “proteins” by performing proteolytic digestion, but why? What else could they be? Performing amino acid sequencing of specific Western blot bands is **biochemically impossible.** Seeing a Western band does not in any way indicate purity: there are many different proteins in this molecular mass range.

Complement your analysis with FISH of proSASS-mRNA locational expression? MERFISH may be a good technology and allows for concomitant protein epitope labelling? Again, siRNA and MERFISH experiments are far beyond the scope of this short descriptive paper. We have neither funding nor personnel to perform these very expensive new animal experiments, nor do we believe they are warranted. **The entire idea of PlosOne is to improve and publish the data that are currently available.**

Regarding antibody specificity, the reviewer fails to appreciate that the authors have decades of experience using this same proSAAS antiserum in tissues and cells, both for staining (**Mitias et al 2024**) as well as for Westerns (**Jarvela et al 2020;** **Shakya et al 2020; Mitias et al 2024**). We have mapped the known epitope for this and all proSAAS antisera used in the paper within the proSAAS sequence (Figure 6). ProSAAS-overexpressing cells exhibit increased intensity of the 21 kDa and 17 kDa bands by Western blotting (**Shakya et al, 2020**). To further persuade the reviewer that we have indeed adequately validated our antisera, we have added a new section regarding antibody specificity to the Methods**:**

“Regarding antibody specificity, the LS46 antiserum has been shown to react with recombinant 21 and 27 kDa proSAAS as well as with the tissue equivalents of these forms (**Shakya et al 2020, Hoshino et al 2011**). The LS46 antiserum also reacts with an endogenous 17 kDa proSAAS fragment (**Shakya et al 2020**) which is present in, and released from, hippocampal synapses upon depolarization (**Mitias et al 2024**).

The proSAAS 1e9 antiserum was obtained from Millipore-Sigma, and was generated in rabbits against a GST-conjugated, proSAAS-derived internal peptide: DGPAGPDAEEAGDETPDVDPELLRYLLGRILAGSADSEGVAAPRRLRRAADHDVGSELPPEGVLGALLRVKRLETPAPQVPARRLLPP. Company Western blot data of proSAAS-transfected HEK cells show that this antiserum reacts with a single band of intact proSAAS which does not appear in untransfected cells; neural and endocrine cells, known to proteolytically process proSAAS (**Sayah et al 2002, Fricker et al 2000**), were not tested. Our tissue data show that this commercial antiserum has similar reactivity to LS46 in reacting with full-length, 21 kDa, 17 kDa and 13 kDa forms of proSAAS (Figure 6 and unpublished data).”

- A general comment to all of your ICC pictures. In general, I do believe that pictures could be of higher quality and magnitude. It is difficult to generate an impression at the cellular level. We disagree and note that the other reviewer did not comment on this and so presumably also agrees with us that the images are satisfactory.

- In figure 2, 3 and 4: Substantial background corrected fluorescens in the INL that almost seem identical to the IPL and GCL in all ICC figures. This somehow contrasts with the findings in Figure 1B showing none-to-mild translation of proSAAS in bipolar cells**.** We do not understand this criticism nor how to address this experimentally. Differences between proSAAS protein and transcript distribution might be due to translocation of the protein to different layers, since neurons have processes that extend far beyond the cell body. We believe we have adequately addressed possible technical reasons (non-specific primary or secondary binding as well as autofluorescence) in the paper ; no-primary controls are now included in the Supplement.

- In figure 2A and B: As you have based your statistical analysis on 22 slices stained with the LS46-antibody and 6 with the 1e9 antibody, it would be appropriate to show representative pictures of both stainings. All IHC figures have now been labeled to identify the specific proSAAS antisera used. Images in Figures 3 and 4 can be used to compare LS46 and 1E9 staining.

How have the retinal slices been selected for analysis? At random?  Based on specific location from the optic nerve head?    We have now added the following statement to the Methods: “Slices were selected only for layer integrity, not for any other properties.”

Why not conduct two separate analyses stratified on the antibodies used to see if the fluorescens patterns are the same or depend on the specific antibodies used? Why this asymmetric usage of antibodies-stained in your analyses i.e., 22 vs 6 (slices)? This issue has now been clarified in the Methods: **“**At least 3 random fields were imaged for each marker gene discussed.” Re asymmetric usage: various marker antibodies are only available as rabbit IgGs; thus, we had to use commercial goat anti-proSAAS antibody for IHC compatibility, or the experiments could not have been performed. This is not unusual.

Could it be an idea to supplement these data with a) a positive control i.e., hippocampal sections as you do in the western blots to assess whether the fluorescens signal likewise confines to the axons and synaptic regions, and b) proper negative controls as I previously mentioned. We disagree that inclusion of hippocampal controls is necessary in this retinal paper. Why? Because we have already published hippocampal “positive control” data using this very same antibody (**Mitias et al 2024**). The reviewer is directed toward this paper, which is multiply referenced in the manuscript.

- It is confusion that you use several synonyms for the same antibody i.e., Abcepta ALS16157 and 1E9. Please use consistent terminology. The reviewer misunderstands; these are two different proSAAS antisera. We used the Abcepta goat proSAAS antiserum for certain IHC experiments because we needed a different species for immunohistochemical compatibility. The Millipore-Sigma 1E9 proSAAS antiserum is a mouse antiserum. This was stated, but is now clarified further in the Methods. We have now added all proSAAS antiserum epitopes to the diagram in Figure 6A.

- Regarding figure 3 and 4: Which ProSAAS antibody have been used? The specific proSAAS antibody used has now been given in all figure legends.

- Figure 4B-D: Please provide full-pictures of the Western blots in order to give the reader an impression of the overall antibody specificity – especially for figure 4C. Why have there not been any pictures provided of western blots using the 1E9 antibodies in figure 4D? We have now provided full pictures of the Western blots for the Supplement. Figure 4 (now Figure 5) has no Western blots. The former Figure 5 (now Figure 6) has all of the Western blots, and Figure 6D (formerly 5D) DOES contain an image of a Western blot using the 1E9 antibody, so we do not understand this criticism.

- The authors claims in the abstract as well as in the introduction that the retina consists of 8 layers. I guess this should be corrected to 10 according to consensus view on the retinal anatomy. Otherwise please argue why. There is disagreement in the literature as to how many “layers” the retina contains. For the purposes of this paper, we have used the 8 layers described in the Kolb et al comprehensive study (40). The other reviewer (presumably an eye expert) had no comment on this issue.

- In section one of the introduction the reader is introduced to the term “proteostasis dysregulation”. Please define accordingly. As requested, we have now defined this phrase: “a disorder in which misfolded proteins contribute to cellular disease”.

- In section one of the introduction the reader is introduced to the neuroretina, please define accordingly. We have added the following language: **“**[neuroretina], the functional neural system responsible for conversion of light into nerve impulses.”

- In section two of the introduction the authors make the following claim “Interestingly, while beta amyloid plaques are present in the aging brain and are especially prominent in Alzheimer’s disease (AD), there is a lack of amyloid plaque development in the retina, despite high concentrations of soluble amyloid precursor protein”. Please cite accordingly for this claim. We have now added additional references to support this statement.

However, I do not believe that the most recent literature on Alzheimer’s manifestations in the retina support this claim. As such, β-amyloid beta plaques have been demonstrated in the retinas of diseased patients with none to severe symptoms, and thus may find implications in the clinic as a biomarker. Please see the following review: Wang L, Mao X. Role of Retinal Amyloid-β in Neurodegenerative Diseases: Overlapping Mechanisms and Emerging Clinical Applications. Int J Mol Sci. 2021 Feb 26;22(5):2360. doi: 10.3390/ijms22052360. PMID: 33653000. We have now added this review to our citations and further discussed this issue; however, we note that AD is not the primary subject of this paper.

- In section three of the introduction the authors makes the following statement “While numerous studies support the theory that proSAAS is a proteostatic stress-reducing chaperone, few studies have examined retinal proSAAS expression”. Please cite the studies mentioned and inform the reader what your study contributes to the literature that these other studies don’t. We have now added references and altered the last line of the Introduction: “ProSAAS is an extracellularly secreted protein that is believed to be a proteostatic stress-reducing chaperone (19), and is associated with synaptic homeostasis (10, 13), but no analyses of proSAAS retinal expression and distribution has yet been performed. In the current report, we evaluated proSAAS expression, location, and molecular forms in the mouse and human retina. “

- Study limitations not discussed in the “Discussion” section. We believe we have presented sufficient study limitations in the Discussion section. Our paper is not intended to be a comprehensive study of proSAAS function, but the first report of proSAAS presence and distribution in the retina.

- Figure 4A: A two-way ANOVA is not the correct statistical test to use. Given that you are comparing the CTF (one dependent variable) across more than two (unpaired) groups defined by a single factor (epitope labelling), a One-Way ANOVA with posthoc comparison and correction should be employed depending on the parametric distribution of your retina. Moreover, what does the vertical bars represent? In figure 4A, please set the y-scale to start at 0. Have there also been conducted a Two-Way Anova in figure 4B?  The reviewer is correct; a one-way ANOVA is the appropriate statistical test for (former) 4A (but not former 4B), as the data are not normally distributed. We re-ran the statistics using this test and the effect significance improved. The paper now incorporates these new statistics for this one experiment (now Figure 5). We have now set the y scale to 0 in panel A as requested.

- In Figure 2B: What kind of unpaired t-test has been used? Why haven’t there been performed statistical comparisons across other groups? We used Student’s t-test ONLY to compare the GCL fluorescent signal to all other layers, ie not to compare layers to each other, thus, this is the appropriate statistical test to use.
